# Assessment of Electronic Health Record for Cancer Research and Patient Care Through a Scoping Review of Cancer Natural Language Processing

**DOI:** 10.1200/CCI.22.00006

**Published:** 2022-08-02

**Authors:** Liwei Wang, Sunyang Fu, Andrew Wen, Xiaoyang Ruan, Huan He, Sijia Liu, Sungrim Moon, Michelle Mai, Irbaz B. Riaz, Nan Wang, Ping Yang, Hua Xu, Jeremy L. Warner, Hongfang Liu

**Affiliations:** ^1^ Department of Artificial Intelligence and Informatics, Mayo Clinic, Rochester, MN; ^2^ Department of Hematology/Oncology, Mayo Clinic, Scottsdale, AZ; ^3^ Department of Computer Science and Engineering, College of Science and Engineering, University of Minnesota, Minneapolis, MN; ^4^ Department of Quantitative Health Sciences, Mayo Clinic, Scottsdale, AZ; ^5^ School of Biomedical Informatics, The University of Texas Health Science Center at Houston, Houston, TX; ^6^ Departments of Medicine (Hematology/Oncology), Vanderbilt University, Nashville, TN; ^7^ Department Biomedical Informatics, Vanderbilt University, Nashville, TN

## Abstract

The advancement of natural language processing (NLP) has promoted the use of detailed textual data in electronic health records (EHRs) to support cancer research and to facilitate patient care. In this review, we aim to assess EHR for cancer research and patient care by using the Minimal Common Oncology Data Elements (mCODE), which is a community-driven effort to define a minimal set of data elements for cancer research and practice. Specifically, we aim to assess the alignment of NLP-extracted data elements with mCODE and review existing NLP methodologies for extracting said data elements.

**METHODS:**

Published literature studies were searched to retrieve cancer-related NLP articles that were written in English and published between January 2010 and September 2020 from main literature databases. After the retrieval, articles with EHRs as the data source were manually identified. A charting form was developed for relevant study analysis and used to categorize data including four main topics: metadata, EHR data and targeted cancer types, NLP methodology, and oncology data elements and standards.

**RESULTS:**

A total of 123 publications were selected finally and included in our analysis. We found that cancer research and patient care require some data elements beyond mCODE as expected. Transparency and reproductivity are not sufficient in NLP methods, and inconsistency in NLP evaluation exists.

**CONCLUSION:**

We conducted a comprehensive review of cancer NLP for research and patient care using EHRs data. Issues and barriers for wide adoption of cancer NLP were identified and discussed.

## INTRODUCTION

As a real-world data source, electronic health records (EHRs) have the potential to provide the comprehensive and relatively timely clinical information necessary to facilitate cancer research and patient care. One of the major challenges associated with the use of EHR data for cancer research and patient care is data quality.^1^ While ideally, all data elements necessary for cancer research and patient care purposes would be rendered accessible in a structured and standardized manner such that no additional efforts would be required to make use of the information contained therein, such is unfortunately not currently the case. For many current usages in so far as cancer research and patient care, the structured data provisioned as part of many popular EHR systems are considered to be incomplete^2^ in that it is limited to specific subsets of clinical data, such as billing codes, and laboratory tests. Some data critical for cancer research and patient care may be recorded only in unstructured text, for example, whether and when a cancer improves or worsens after a given therapy.^3^ Advancement in natural language processing (NLP) techniques has promoted the usage of clinical information extraction (IE) from unstructured texts to help supplement this information gap,^4,5^ and consequently, the application of NLP in cancer domain has also been increasing.

**CONTEXT**

**Key Objective**
To assess electronic health record (EHR) for cancer research and patient care through assessing the coverage of natural language processing (NLP)–derived data elements by the Minimal Common Oncology Data Elements and reviewing existing NLP methodologies for data extraction.
**Knowledge Generated**
A comprehensive review of cancer NLP for research and patient care using EHRs data extraction was conducted. Issues and barriers for wide adoption of cancer NLP were identified and discussed.
**Relevance**
Overcoming the identified issues and barriers will improve the readiness of EHRs for cancer research and patient care, thus propelling translational clinical research and care.

To gain an understanding of the gaps and opportunities of NLP in EHR for cancer research and patient care, we conducted a scoping review of literature relevant to cancer NLP in EHR. We hypothesize that the need of NLP solutions to extract data elements reflects critical information not captured by structured EHR, and the readiness of NLP for extracting those data elements highly depends on the performance of NLP methodology involving many aspects including NLP tools, methods, evaluation, and reproducibility. Data elements defined as part of the Minimal Common Oncology Data Elements (mCODE) standard are used as a proxy for data elements that would be important for cancer research and patient care.^6^ The mCODE data standard was initiated from 2018 by ASCO, other founding collaborators, and a group of collaborators, including oncologists, informaticians, researchers, and experts in terminologies and standards, to develop and maintain standard computable oncology data formats in EHR for cancer research and practice. The final mCODE data standard (version 1.0) included six primary groups (domains): patient, disease, laboratory/vital, genomics, treatment, and outcome. Each domain is organized into several concepts, which then have associated data elements. These concepts are referred to as profiles. In total, 23 profiles exist across mCODE's six primary domains (Appendix Table A1). These data elements are linked to standard coding systems such as American Joint Committee on Cancer,^7^ ClinVar,^8^ International Classification of Diseases (10th revision), and Clinical Modification.^9^

Some prior work exists. In 2016, Yim et al conducted a similar literature review,^10^ providing an introduction to NLP and its potential applications in oncology, describing specific tools available, and summarizing on the state of the current technology with respect to cancer case identification, staging, and outcomes quantification. Similarly, in 2019, Datta et al conducted a scoping review of clinical NLP literature extracting information from cancer-related EHR notes according to frame semantic principles.^11^ They created frames from the reviewed articles pertaining to cancer information such as cancer diagnosis, tumor description, cancer procedure, breast cancer diagnosis, prostate cancer diagnosis, and pain in patients with prostate cancer. This review paper emphasized data model construction. Another relevant work reviewed the major NLP algorithmic advances and cancer NLP application developments over 3 years since 2016, summarizing the main trends of clinical cancer phenotype extraction from EHRs.^12^

In our scoping review, we focus on (1) presenting all the efforts using NLP in extracting cancer information as an end point or intermedium step and aligning them to mCODE, that is, the new data standard for oncology domain and (2) categorizing them based on NLP methodology.

## METHODS

This scoping review was performed based on the following five stages of the framework from Arksey and O'Malley.^13^

### Identifying the Research Question

In this scoping review, we aim to assess the alignment of NLP-extracted data elements with mCODE and review existing NLP methodologies for extracting said data elements.

### Identifying Relevant Studies

We included articles to a 10-year period from January 1, 2010, to September 4, 2020. Only studies written in English were considered. Literature databases surveyed included Ovid MEDLINE(R) and Epub Ahead of Print, In-Process & Other Non-Indexed Citations, and Daily; Ovid Embase; Ovid Cochrane Central Register of Controlled Trials; Ovid Cochrane Database of Systematic Reviews; Scopus; and Web of Science. The search strategy for articles using NLP in cancer domain was designed and conducted by an experienced librarian (Larry J. Prokop). A detailed description of the search strategies used is provided in Appendix 1.

### Study Selection

All the titles and abstracts after deduplication were screened, and the publications were included if

1. NLP was conducted for cancer, as defined below.
  - The NLP involved could be used either as an end product or an inter-medium step for other downstream analytics.
  - The NLP was cancer related.
2. EHR-sourced textual data in English were used as data source.

We excluded publications if they were

1. Not written in English.
2. Retrieved by irrelevant term matching.
3. Using non-EHR data sources such as literature, web resources, knowledge bases, clinical trials, and clinical guidelines.
4. Not using English EHR data.
5. Review papers/letters.

### Charting the Relevant Studies

A standardized charting form was established to synthetize relevant publications. The information of interest can be categorized into four main sections: metadata, EHR data and targeted cancer types, oncology data elements and standards, and NLP methodology.

**FIG 1.**
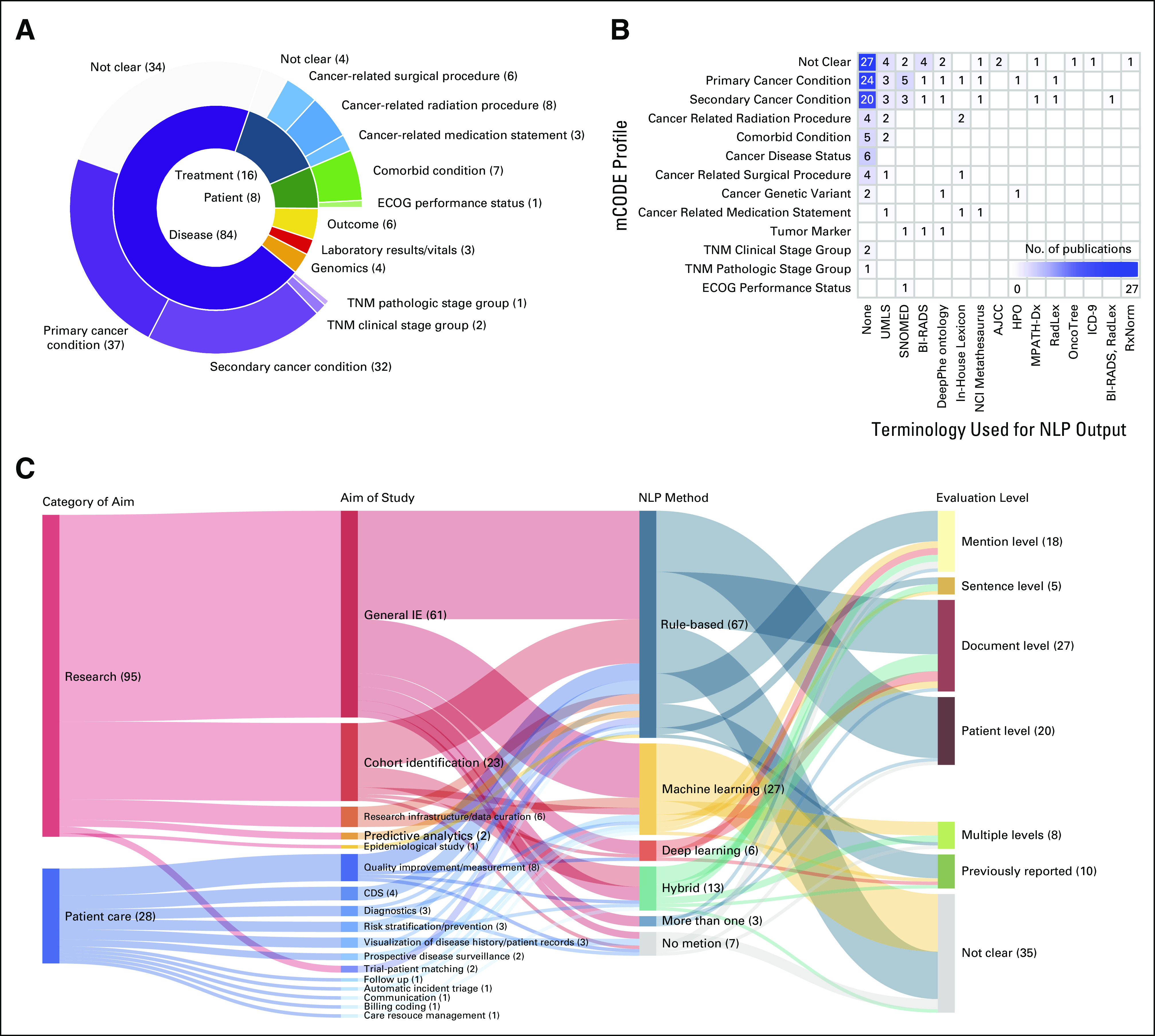
Synthetic analysis for mCODE and NLP methodology. (A) Distribution of data elements covered by mCODE. (B) Clustering visualization of the mCODE profiles and standardized terminologies. (C) Synthetic analysis for NLP methods, study aim, and evaluation level. AJCC, American Joint Committee on Cancer; BI-RADS, Breast Imaging Reporting and Data System; CDS, clinical decision support; ECOG, Eastern Cooperative Oncology Group; HPO, Human Phenotype Ontology; ICD-9, International Classification of Diseases (9th revision); IE, information extraction; mCODE, Minimal Common Oncology Data Elements; MPATH-Dx, Melanocytic Pathology Assessment Tool and Hierarchy for Diagnosis; NCI, National Cancer Institute; NLP, natural language processing; RadLex, Radiology Lexicon; RxNorm, no full name; UMLS, Unified Medical Language System.

The Metadata section consisted of publication year; publication domain; major country of authors (first/senior author); types of author's organizations; the main study aim, defined as one of research and patient care, of the article; and the NLP study aim. The NLP study aim was classified into two groups: IE as an end point and IE as the input for machine learning. The research aim and patient care aim were further subcategorized, and more details are presented in Figure 1C.

The EHR Data and Targeted Cancer Types section aimed to summarize information including the targeted cancer types of the article, data time frame, and document types (eg, clinical notes, pathology report, and radiology reports). We defined the targeted cancer types using the ultimate cancer type around which the study was focused, for example, if the study focused on lung nodules but the aim of this study was to screen lung cancer, then we charted the targeted cancer type as lung cancer.

In summarizing oncology data elements and standards, we aggregated NLP-extracted data elements based on the 23 profiles presented in Appendix Table A1. Additionally, we also examined the standardized terminology used for any normalization done of the NLP output for these data elements.

In the NLP methodology section, we described the most frequently used NLP tools, frameworks, or toolkits and cancer-specific NLP tools. In addition, we also analyzed NLP methods, the evaluation environment, performance metrics used, NLP methods evaluation granularity, and the study's reproducibility and rigor of evaluation.

NLP methods were categorized into one of six groups: rule-based, machine learning, deep learning, hybrid (one model with different approaches, eg, rules and machine learning), more than one (multiple models), and unspecified. Evaluation granularities included mention level, sentence level, document level, patient level, multiple levels, previously reported, and unknown.

To understand the study's evaluation scope (internal *v* external), we categorized the evaluation environment into one of the following groups: single center, multiple centers, Veterans Affairs, benchmark data set, single center and benchmark, no evaluation, and unspecified.

In many NLP studies, trust and adoption of any study outcomes are dependent on the validity and reproducibility of the NLP methods used. As such, in this review, we assessed the reporting patterns for NLP methods and evaluation methodologies. We first examine the reproducibility of NLP methods by evaluating code sharing, a crucial component of transparent and reproducible NLP research^14^ (Table 1). To assess the rigor of evaluation, we considered four major evaluation best practices^5^ (Table 1). Specifically, each publication is defaulted to have a score of 4, from which 1 point is subtracted whenever one of the criteria is met. Therefore, the maximum score for rigor of evaluation is 4 while the minimum is 0.

**TABLE 1.**
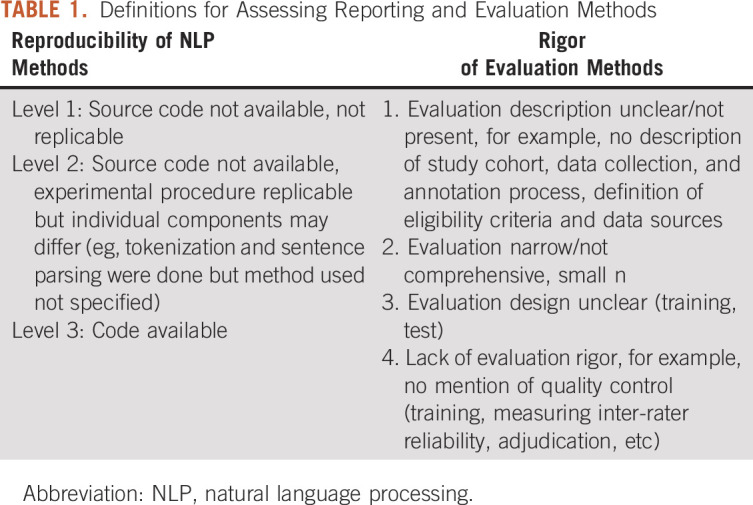
Definitions for Assessing Reporting and Evaluation Methods

Each reviewer is responsible for charting 2-4 aspects and checking the charting quality of other reviewers by randomly sampling 10% of the data in the easily charted case or otherwise fully reviewing all data. When charting results disagreed between individual reviewers, reviewers met to resolve uncertainties.

### Collating, Summarizing, and Reporting the Results

The results from the data charting were summarized, analyzed, and visualized both within and across the sections to present an overview of the scope of the application of NLP in cancer domain.

## RESULTS

**FIG 2.**
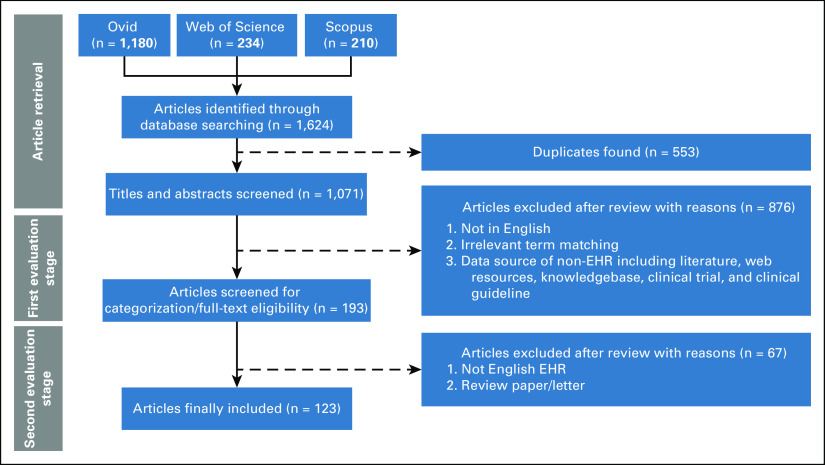
Overview of article selection process. EHR, electronic health record.

Figure 2 shows the article selection process. Finally, a comprehensive full-text review of the resulting 123 studies was performed by the study team. All data extractions from the articles (charting items) are presented in the Data Supplement.

### Metadata

**FIG 3.**
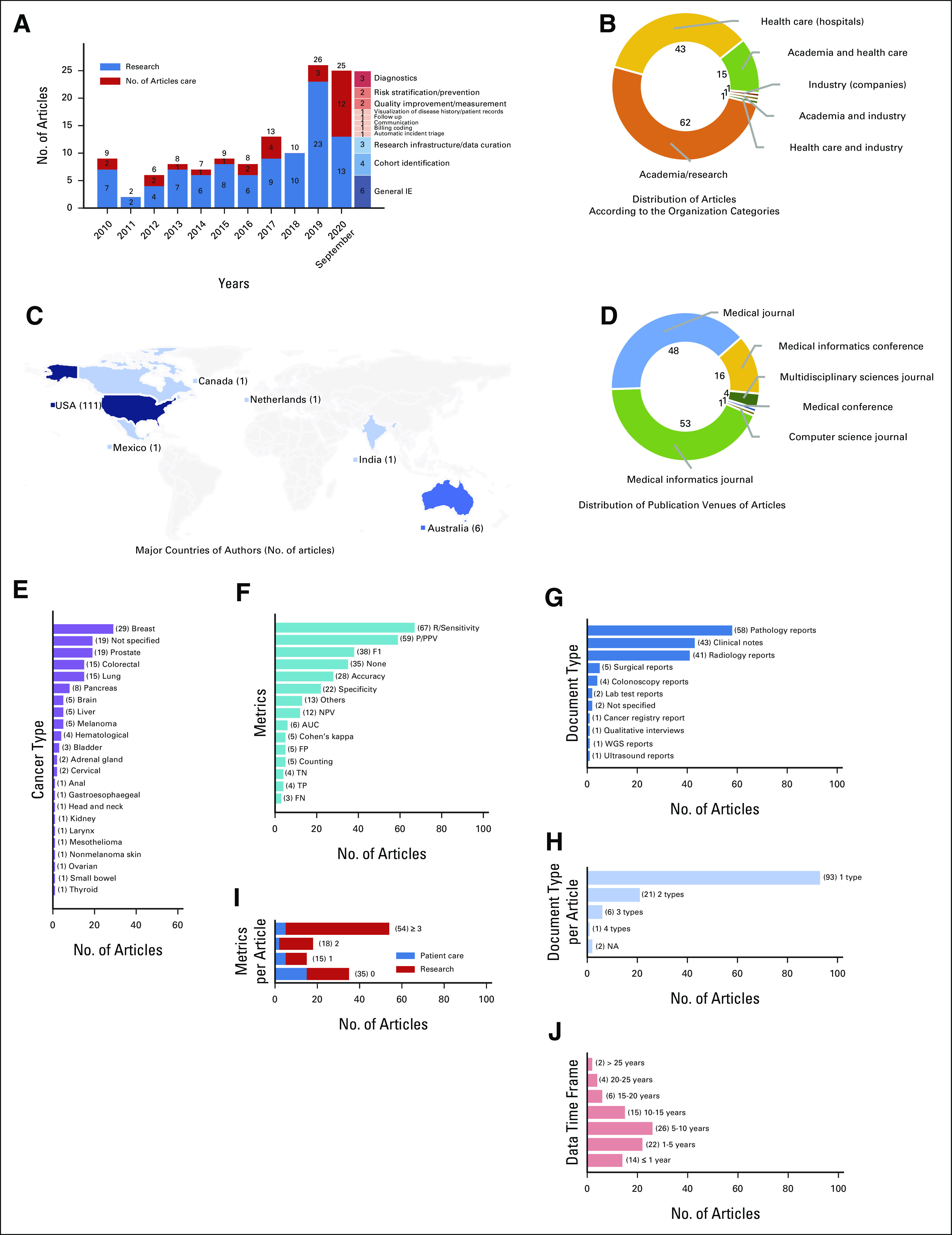
Analysis of metadata, EHR data scope, and evaluation metrics of included articles. (A) Distribution of articles over years. (B) Distribution of articles according to the organization categories. (C) Countries of major authors (No. of articles). (D) Distribution of publication venues of articles. (E) Distribution of cancer type. (F) Distribution of metrics. (G) Distribution of document type. (H) Histogram of document type number. (I) Histogram of metric number. (J) Histogram of data time frame. AUC, area under the curve; EHR, electronic health record; F1, no full name; FN, false negative; FP, false positive; IE, information extraction; NA, not applicable; NPV, negative predictive value; PPV, positive predictive value; TN, true negative; TP, true positive; WGS, Whole Genome Sequencing.

Figure 3A presents the distribution of articles, stratified by study aim and year, showing an increase in research interest for NLP in cancer. As shown in the author-country distribution depicted in Figure 3C, 111 articles (90%) had major authors from the United States while six (5%) had major authors from Australia. Figure 3B shows the distribution of articles according to the organization categories of the respective authors. In terms of publication venue, medical informatics or medical journals were the main venues for related studies, as shown in Figure 3D. In addition, NLP was used for IE as an end point in 101 articles and as machine learning input in 22 articles.

### EHR Data and Targeted Cancer Types

#### Document types.

Of the reviewed studies (Fig 3G), 58 (47%) studies extracted information from pathology reports,^15-72^ 43 (35%) studies from clinical notes,^3,20,28,30,35,36,46,61,64,65,70,72-103^ and 41 (33%) studies from radiology reports.^20,36-38,43,46,48,52,61,62,70,76,89,104-131^ As various document types could be used for one study, document type numbers in each article were further analyzed (Fig 3H).

#### Targeted cancer types.

The cancer types of interest for our reviewed studies were scattered across a wide spectrum, and significant variability was present in the number of articles for each cancer type of interest (Fig 3E). Of note, a single study may involve multiple cancer types. Among the 22 cancer types specified across all reviewed articles, breast cancer was the most intensively studied, being the primary cancer type of interest for 29 articles (24%), followed by prostate, colorectal, and lung cancers at 19 (15%), 15 (12%), and 15 (12%), respectively.

**FIG A1.**
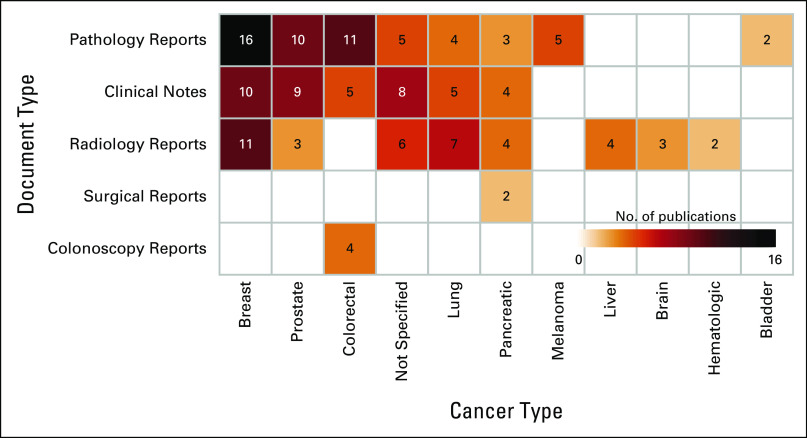
A heatmap of document types and targeted cancer types. Those with < 2 publications not shown.

A clustering can be visualized for the document types and targeted cancer types (Appendix Fig A1). Pathology reports, clinical notes, and radiology reports were the major data sources for extracting information related to breast, lung, liver, prostate, colorectal, brain, pancreatic, melanoma, head, and neck cancers.

#### Data time frame.

In terms of the time frame of the data used for NLP, we calculated the age in years of the data used after excluding 33 studies that did not specify a data time frame. Before the 10-year mark, the number of studies increased as data age increased. Conversely, past the 10-year mark, the number of studies decreased as data age increased. We hypothesize that this trend may reflect the availability of EHR data (Fig 3J).

### Oncology Data Elements and Standards

We first analyzed the 106 articles extracting data elements that can be mapped to a mCODE (Fig 1A). The distribution of data elements extracted was imbalanced across all six mCODE groups, with coverage extending to 12 of the 23 mCODE profiles (Appendix Table A1). Note that there are 31 articles extracting data elements corresponding to more than one mCODE group.

The most studied mCODE group was disease with 84 unique studies, associated with mCODE profiles of primary cancer condition, secondary cancer condition, TNM clinical stage group, and TNM pathologic stage group (Fig 1A). Our review revealed that most studies recorded no obvious differentiation between clinical and pathologic staging, and it was therefore difficult to categorize studies under these profiles as mCODE requires. As such, TNM staging was labeled as not clear under the disease group. In addition, those data elements without clear indications as to the primary cancer or secondary cancer conditions involved were similarly labeled as not clear. The second most studied mCODE group was treatment with 16 unique studies, followed by the patient, outcome, genomics, and laboratory/vital groups with 8, 6, 4, and 3 corresponding studies, respectively.

There are 20 articles extracting data elements not covered by mCODE. Table 2 presents the statistics of these data elements. Certain cancer screening criteria and social determinants of health were the two areas with data elements outside of mCODE's scope. For those 20 articles, only five used standard terminologies to code NLP output, including the Thyroid Imaging Reporting & Data System ^131^ and the Unified Medical Language System.^54,90,100,102^

**TABLE 2.**
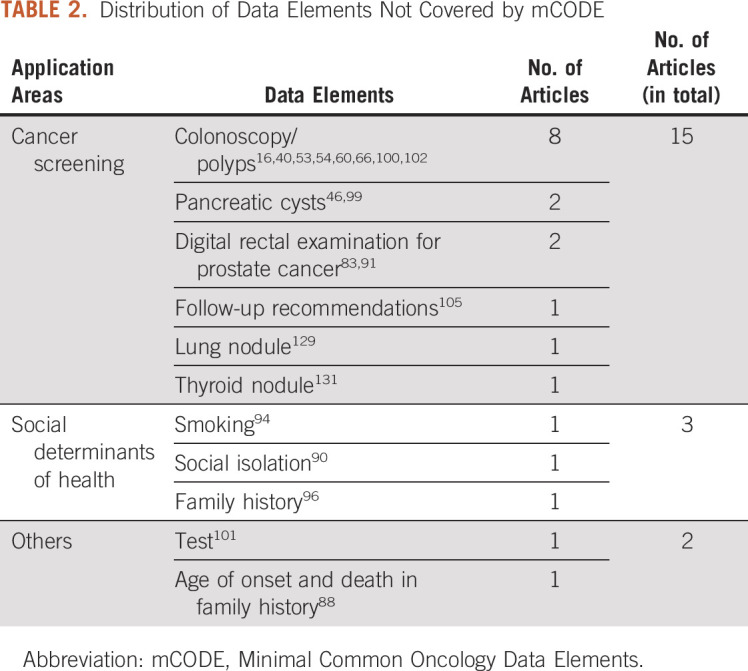
Distribution of Data Elements Not Covered by mCODE

**FIG A2.**
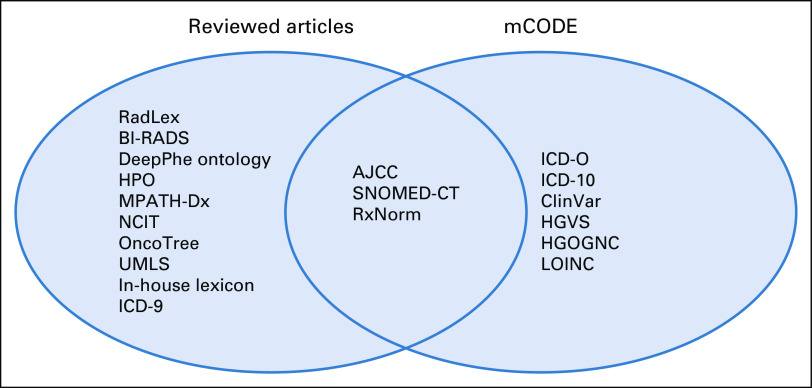
Comparison of standardized terminologies for data elements between the reviewed articles and mCODE. AJCC, American Joint Committee on Cancer; BI-RADS, Breast Imaging Reporting and Data System; HGOGNC, Human Genome Organization Gene Nomenclature Committee; HGVS, Human Genome Variation Society; HPO, Human Phenotype Ontology; ICD-9, International Classification of Diseases (9th revision); ICD-10, International Classification of Diseases (10th revision); ICD-O, International Classification of Diseases for Oncology; LOINC, Logical Observation Identifiers Names and Codes; MPATH-Dx, Melanocytic Pathology Assessment Tool and Hierarchy for Diagnosis; NCIT, National Cancer Institute Thesaurus; RadLex, Radiology Lexicon; RxNorm, no full name; SNOMED-CT, SNOMED Clinical Terms; UMLS, Unified Medical Language System.

About one third of the studies (43 of 123) adopted standard terminologies to normalize NLP output. Appendix Figure A2 shows a comparison between the reviewed articles and mCODE regarding adopted standard terminologies.

Figure 1B shows the clustering visualization of the mCODE profiles and standardized terminologies. A significant portion of reviewed studies failed to adopt standards for NLP output, while those profiles under the disease group were the major mCODE profiles normalized. The Unified Medical Language System covered the most profiles, followed by SNOMED.

### NLP Methodology

#### NLP tools.

Table 3 presents cancer-specific NLP tools, and the most frequently used general NLP tools, frameworks, or toolkits, which are consistent with our previous review.^4,5^

**TABLE 3.**
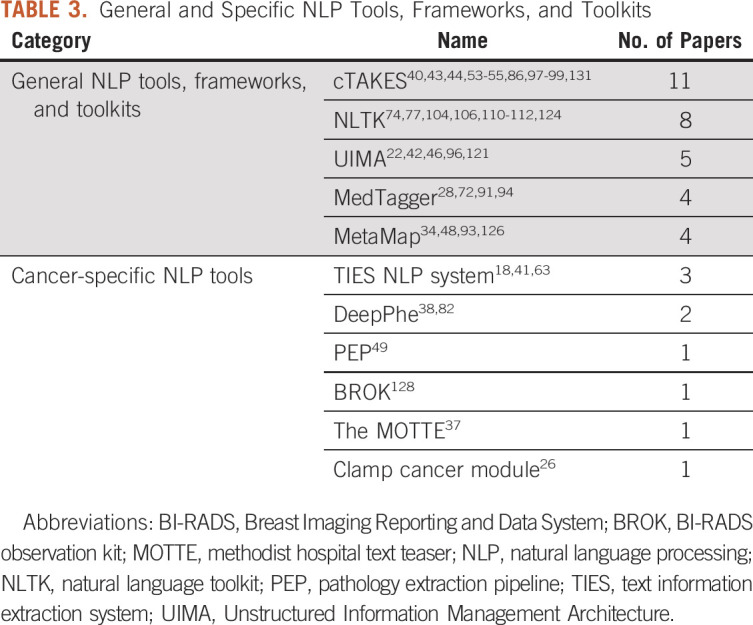
General and Specific NLP Tools, Frameworks, and Toolkits

#### NLP methods and synthetic analysis.

**FIG A3.**
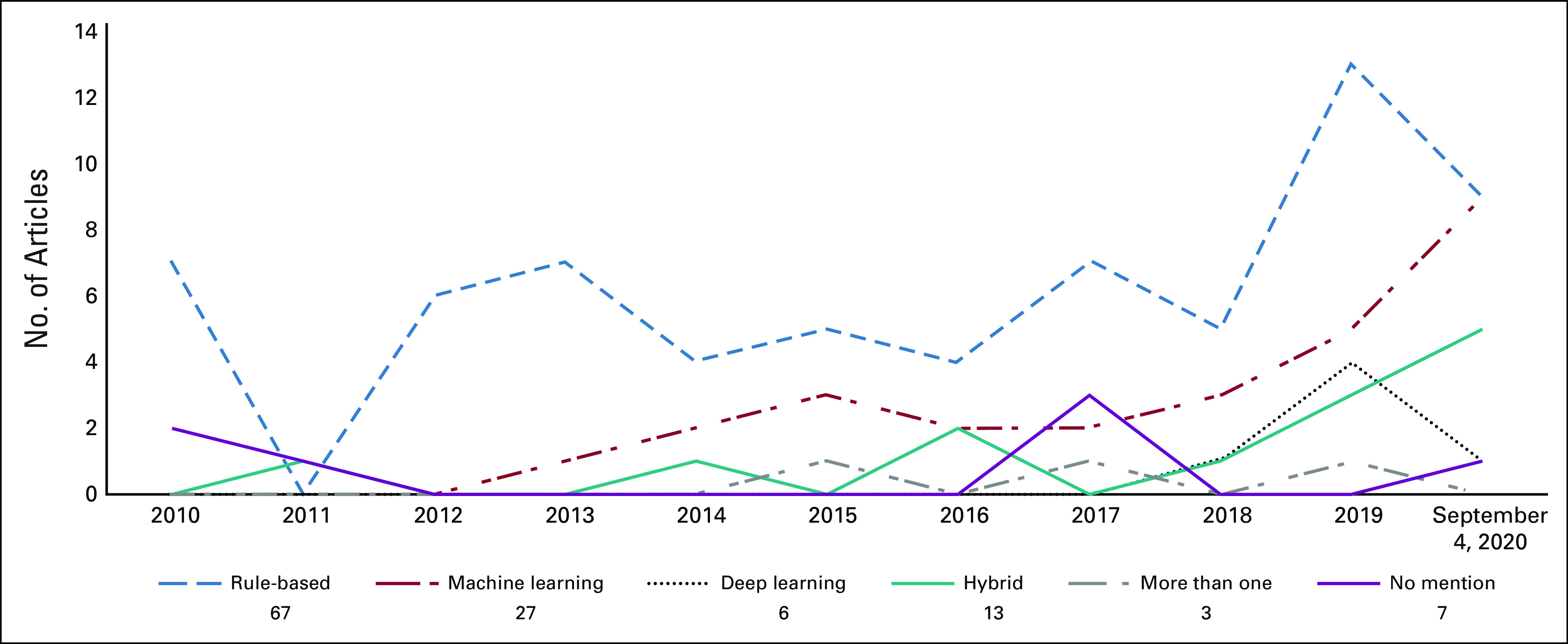
Analysis of NLP methods. NLP, natural language processing.

Appendix Figure A3 shows the changing trend of various NLP methods over time and the total number for each method. Rule-based methods are currently predominant. There is, however, an increasing trend in adoption of machine learning and hybrid methods. Despite the recently increasing adoption of deep learning methods^132^ for NLP in the general domain, a delay of such application in the cancer domain can be observed.

To delineate the association among the charted items, we conducted a synthetic analysis for NLP methods, study aim of the article, and evaluation level (Fig 1C).

#### Evaluation setting.

Among the total 123 articles, 86 (70%) articles conducted the NLP evaluation in a single-site environment, 14 (11%) articles conducted a multisite evaluation, two (2%) studies used an external benchmark data set (eg, i2b2 shared task, Medical Information Mart for Intensive Care database), and four (3%) studies were based on VA data. In addition, 10 (8%) studies did not specify an evaluation environment, and seven (6%) studies did not report evaluation details.

#### Performance metrics.

A variety of metrics were used to evaluate NLP methods (Fig 3F). The lack of consistency in performance evaluation makes cross-comparison of NLP systems difficult. Among the 35 articles reporting no evaluation metrics of NLP methods, 20 articles had the study aim for research, accounting for 21% of articles for research, and 15 were for patient care, accounting for 56% of this category (Fig 3I). In general, articles for research purpose reported more NLP evaluation metrics compared with those for patient care (Fig 3I).

#### Reproducibility and evaluation rigor.

For the ability to replicate NLP methods, there were 78 articles (63%) in level 1. Level 2 contained 35 articles (28%), and level 3 had only 10 articles (8%). For the evaluation rigor of NLP methods in each study (defined in Table 1), 21 (17%) articles were rated as 0, 24 (20%) articles rated as 1, 32 (26%) rated as 2, 41 (34%) rated as 3, and five (4%) rated as 4.

## DISCUSSION

The rapid growth of dense longitudinal EHR data sets provides substantial opportunities for the application of NLP in the cancer domain in recent years, as evidenced by the increased article count. It is extremely encouraging to see a jump of the NLP applications with a patient care (as opposed to research) focus in 2020 (12 articles). This growth reflects the value of NLP for clinical practice as NLP becomes more accessible. In the meanwhile, issues and barriers for wide adoption of cancer NLP were identified and discussed as follows.

NLP-targeted cancer types have covered most of the common cancer types collected by Cancer Stat Facts of the National Cancer Institute.^133^ Cancer is highly complex and diverse; consequently, cancer research and patient care require diverse types of data, which can be reflected in our document type summary (Fig 3G).

The high utilization of NLP to extract information from pathology reports, clinical notes, and radiology reports demonstrates that important data elements for cancer research and patient care were embedded in text. Even with the ongoing efforts of standardizing pathology and radiology reporting,^134,135^ the actual implementations seem to be insufficient.

mCODE is a current standard for EHR data to represent essential clinical elements for cancer patients, benefiting both oncology researchers and providers. We observed an imbalanced distribution across the six mCODE groups. Some groups, that is, the genomics group (four studies) and the laboratory/vital group (three studies), primarily come from structured data, thus less involved. Nevertheless, from the perspective of precision oncology and given that genomic data created through molecular diagnostics and treatment have been increasingly accumulated in EHR, tackling genomic data extraction from text could greatly advance the efficient secondary use of EHRs. In the meanwhile, aligning the standardized terminologies for data elements to mCODEs, for example, International Classification of Diseases for Oncology, American Joint Committee on Cancer, and ClinVar, is also important.

NLP for the outcome group (six studies) was under explored probably because of the intrinsic challenge of cancer disease status assessment as it mostly relies on clinicians' qualitative judgment on the current trend of the cancer. The judgment can be based on a single type or multiple kinds of evidence, such as imaging data, assessment of symptoms, tumor markers, and laboratory data, at a given time. Among those six studies, a study by Lee et al^104^ proposed a scalable NLP pipeline that was capable of inferring Brain Tumor Reporting and Data System report scores. In the study by Sevenster et al, measurements used to synthesize treatment response status were extracted and paired across consecutive free-text computed tomography reports.^125^ Clinically relevant outcomes can be extracted by NLP methods based on rules and machine learning^130^ as well as deep NLP models^113^ from radiologic reports. The codependent effects of NLP and machine learning in categorizing cancer disease status were investigated in computed tomography and magnetic resonance imaging reports.^117^ Those studies focused on radiology reports which are ubiquitous and central to ascertainment of cancer disease status. However, additional information relevant to cancer status cannot be captured in radiology reports, such as laboratory results. The only study that did not use radiology reports applied deep NLP models to extract meaningful outcomes from clinical progress notes.^3^

Owing to the complexity of cancer disease status assessment, current EHR recording practice does not favor a seamless outcome assessment. For example, there is generally not a mechanism to input the staging information into the radiation oncology EHR or link metastatic sites to the original diagnosis, which are usually of interest for outcome analyses.^136^ Moreover, oncologists sometimes have irreducible uncertainty about whether the cancer is responding or progressing when a clinical note is filed.^3^ In addition, recorded cancer disease statuses may be time varying resulting in multiple instances of outcomes, which poses additional challenges for outcome extraction. For such mCODE data element as cancer disease status without a discrete data field in EHR, we believe NLP could play the most important role in extracting and structuring it.

Our review does indicate that some critical information elements covered by mCODE and needed for cancer research and patient care are not currently captured as part of structured EHR data. Since mCODE is used only for minimal critical oncology specialty information, it is as expected that those data elements extracted by NLP but not covered by mCODE are also important for cancer research and patient care, ie, cancer screening such as lung nodule screening and social determinants of health such as family history and smoking. This implies a great opportunity for improving structured data capture in EHRs to improve the readiness of EHRs for cancer research and patient care.

Recently, US Food and Drug Administration published a guidance for assessing EHRs and medical claims data to support regulatory decision making for drug and biological products, which also provides insightful and sharable recommendations for real-world data applications in other domains. For AI methods extracting data elements from unstructured data, it recommended to specify methods, tools, data sources, and the metrics associated with validation of the methods. Although the ability to replicate NLP methods was not mentioned in the US Food and Drug Administration guidance, we consider it a crucial factor that affects the adoption of NLP methods in the cancer domain. Unfortunately, our review revealed that more than half of the surveyed articles failed to provide either the source code or sufficient detail in the methodology to fully replicate the developed NLP systems, indicating a poor reporting practice in the domain.

Transparency and sharing contribute to assessment of research reproducibility, robustness, and replicability. Guidance on code sharing aligned with a specific study aim would be helpful to support transparency and reproducibility that would then strengthen the credibility of NLP results and promote downstream patient care leveraging NLP results.

For studies reporting no evaluation metrics for NLP methods, reasons that justify no assessment include that NLP evaluation has been reported in a previous publication or that NLP was simply used as an intermediate feature extraction step that feeds into a downstream machine learning algorithm that was itself evaluated. Although most studies reported metrics for NLP methods, reported metrics were not consistent across all studies, but rather study dependent, even for those with the same study aim.

Most studies inherited traditional and typical NLP study paradigms, focusing on mention-level, sentence-level, or document-level evaluations using a benchmark data set. Patient-level evaluation was relatively sparsely used (20 articles), and such an alignment to real-world clinical settings was very seldom seen. A considerable number of studies assigned with not clear evaluation level were extracting data elements from pathology reports. Without pondering that potential conflicting information could be reported for a single patient through multiple text reports, the evaluation level was failed to be clarified in these studies. There is certainly a need to evaluate NLP methods to an extent where the true level of complexity of clinical EHR data could be reflected following a scientific and rigorous evaluation process,^137^ thus propelling the translation to clinical application.

Although NLP solutions can be potentially leveraged for large-scale IE, single-site studies are still predominant among current cancer NLP research. We acknowledge the potential barriers of multisite cancer NLP research such as complex concept definitions requiring extensive effort for participating sites to reach consensus and variations in clinical documentation patterns and data infrastructures (eg, different extract, transform, and load processes) hampering cross-institutional experimentation. Regardless, we observed that 11% of studies surveyed involved more than one EHR in the study, demonstrating the feasibility of multi-institutional NLP efforts in cancer research and care.

There exist some limitations in our study. First, this review may be biased due to the potential of missing relevant articles caused by search strings and databases selected. Second, we only included articles written in English with the focus on using NLP in cancer EHR. Articles written in other languages would also provide valuable information. Third, our review did not include methods based on non-English EHRs. Finally, our study may also suffer the inherent ambiguity associated with data element collection, normalization, and analysis due to subjectivity introduced in the review process.

## Figures and Tables

**TABLE A1.**
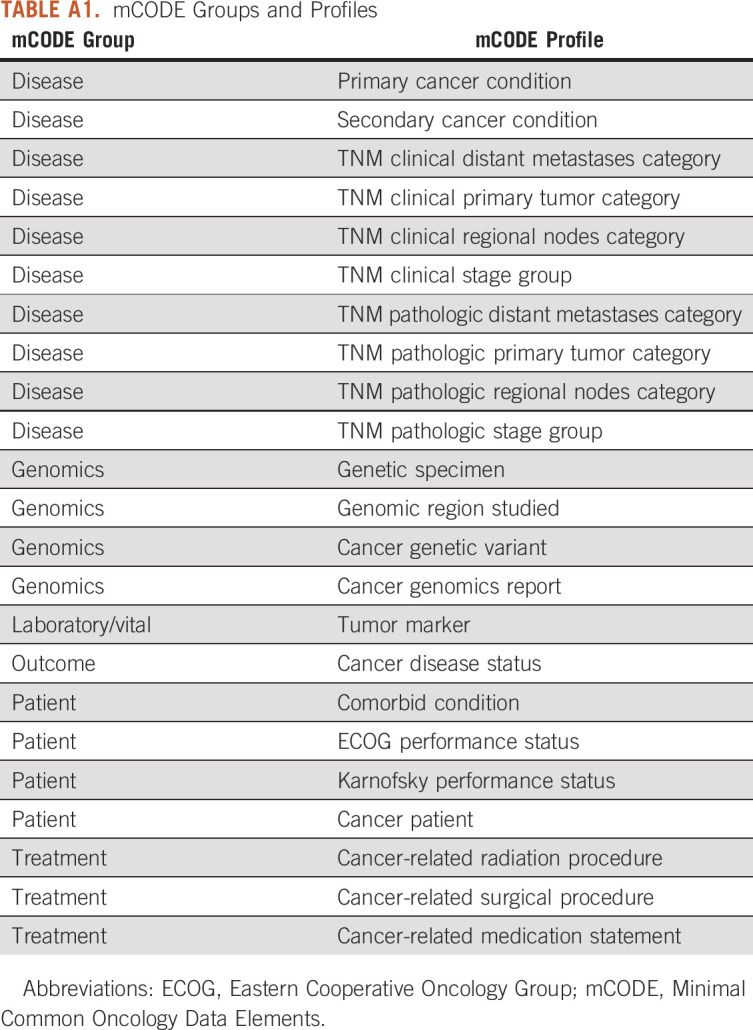
mCODE Groups and Profiles

## APPENDIX 1. SEARCH STRATEGIES

### Ovid

Database(s): EBM Reviews—Cochrane Central Register of Controlled Trials August 2020, EBM Reviews—Cochrane Database of Systematic Reviews 2005 to September 3, 2020, Embase 1974 to 2020 September 4, Ovid MEDLINE(R) and Epub Ahead of Print, In-Process & Other Non-Indexed Citations and Daily 1946 to September 4, 2020.

Search Strategy

### Scopus

1. TITLE-ABS-KEY(“coreference resolution” OR “co-reference resolution” OR “information extraction” OR “named entity extraction” OR “named entity recognition” OR “natural language processing” OR NLP OR “relation extraction” OR “text mining”)
2. TITLE-ABS-KEY((hodgkin* W/1 disease) or adenocarcinoma* or adenoma* or anticarcinogen* or Astrocytoma* or blastoma* or burkitt* or cancer* or carcinogen* or carcinoid* or carcinom* or carcinosarcoma* or chordoma* or “Chronic Myeloproliferative Disorder*” or craniopharyngioma* or ependymoma* or Esthesioneuroblastoma* or germinoma* or “gestational trophoblastic disease*” or Glioblastoma* or glioma* or gonadoblastoma* or hepatoblastoma* or histeocytoma* or histiocytoma* or histiocytos* or leukaemi* or leukemi* or lymphangioma* or lymphangiomyoma* or lymphangiosarcoma* or lymphom* or Macroglobulinemia* or malignan* or melanom* or meningioma* or mesenchymoma* or mesonephroma* or Mesothelioma* or metasta* or “multiple myeloma*” or “Mycosis Fungoide*” or neoplas* or neuroblastoma* or neuroma* or nonmelanoma* or nsclc or oncogen* or oncolog* or ostesarcoma* or Papillomatos* or paraganglioma* or paraneoplas* or pheochromocytoma* or plasmacytoma* or precancerous or retinoblastoma* or Rhabdomyosarcoma* or Sarcoma* or “section 16” or “Szary Syndrome*” or teratocarcinoma* or teratoma* or tumor* or tumor*)
3. LANGUAGE(english)
4. 1 and 2 and 3
5. TITLE-ABS-KEY((alpaca OR alpacas OR amphibian OR amphibians OR animal OR animals OR antelope OR armadillo OR armadillos OR avian OR baboon OR baboons OR beagle OR beagles OR bee OR bees OR bird OR birds OR bison OR bovine OR buffalo OR buffaloes OR buffalos OR “c elegans” OR “Caenorhabditis elegans” OR camel OR camels OR canine OR canines OR carp OR cats OR cattle OR chick OR chicken OR chickens OR chicks OR chimp OR chimpanze OR chimpanzees OR chimps OR cow OR cows OR “D melanogaster” OR “dairy calf” OR “dairy calves” OR deer OR dog OR dogs OR donkey OR donkeys OR drosophila OR “Drosophila melanogaster” OR duck OR duckling OR ducklings OR ducks OR equid OR equids OR equine OR equines OR feline OR felines OR ferret OR ferrets OR finch OR finches OR fish OR flatworm OR flatworms OR fox OR foxes OR frog OR frogs OR “fruit flies” OR “fruit fly” OR “G mellonella” OR “Galleria mellonella” OR geese OR gerbil OR gerbils OR goat OR goats OR goose OR gorilla OR gorillas OR hamster OR hamsters OR hare OR hares OR heifer OR heifers OR horse OR horses OR insect OR insects OR jellyfish OR kangaroo OR kangaroos OR kitten OR kittens OR lagomorph OR lagomorphs OR lamb OR lambs OR llama OR llamas OR macaque OR macaques OR macaw OR macaws OR marmoset OR marmosets OR mice OR minipig OR minipigs OR mink OR minks OR monkey OR monkeys OR mouse OR mule OR mules OR nematode OR nematodes OR octopus OR octopuses OR orangutan OR “orang-utan” OR orangutans OR “orang-utans” OR oxen OR parrot OR parrots OR pig OR pigeon OR pigeons OR piglet OR piglets OR pigs OR porcine OR primate OR primates OR quail OR rabbit OR rabbits OR rat OR rats OR reptile OR reptiles OR rodent OR rodents OR ruminant OR ruminants OR salmon OR sheep OR shrimp OR slug OR slugs OR swine OR tamarin OR tamarins OR toad OR toads OR trout OR urchin OR urchins OR vole OR voles OR waxworm OR waxworms OR worm OR worms OR xenopus OR “zebra fish” OR zebrafish) AND NOT (human OR humans or patient or patients))
6. 4 and not 5
7. DOCTYPE(ed) OR DOCTYPE(bk) OR DOCTYPE(er) OR DOCTYPE(no) OR DOCTYPE(sh)
8. 6 and not 7
9. INDEX(embase) OR INDEX(medline) OR PMID(0* OR 1* OR 2* OR 3* OR 4* OR 5* OR 6* OR 7* OR 8* OR 9*)
10. 8 and not 9

### Web of Science

1. TOPIC: ((“coreference resolution” OR “co-reference resolution” OR “information extraction”OR “named entity extraction” OR “named entity recognition” OR “natural language processing” OR NLP OR “relation extraction” OR “text mining”)) *AND* **TOPIC:** (((hodgkin* NEAR/1 disease) or adenocarcinoma* or adenoma* or anticarcinogen* or Astrocytoma* or blastoma* or burkitt* or cancer* or carcinogen* or carcinoid* or carcinom* or carcinosarcoma* or chordoma* or “Chronic Myeloproliferative Disorder*” or craniopharyngioma* or ependymoma* or Esthesioneuroblastoma* or germinoma* or “gestational trophoblastic disease*” or Glioblastoma* or glioma* or gonadoblastoma* or hepatoblastoma* or histeocytoma* or histiocytoma* or histiocytos* or leukaemi* or leukemi* or lymphangioma* or lymphangiomyoma* or lymphangiosarcoma* or lymphom* or Macroglobulinemia* or malignan* or melanom* or meningioma* or mesenchymoma* or mesonephroma* or Mesothelioma* or metasta* or “multiple myeloma*” or “Mycosis Fungoide*” or neoplas* or neuroblastoma* or neuroma* or nonmelanoma* or nsclc or oncogen* or oncolog* or ostesarcoma* or Papillomatos* or paraganglioma* or paraneoplas* or pheochromocytoma* or plasmacytoma* or precancerous or retinoblastoma* or Rhabdomyosarcoma* or Sarcoma* or “section 16” or “Szary Syndrome*” or teratocarcinoma* or teratoma* or tumor* or tumour*)) *AND* **LANGUAGE:** (English) *AND* **DOCUMENT TYPES:** (Article OR Abstract of Published Item OR Data Paper OR Letter OR Meeting Abstract OR Proceedings Paper OR Review OR Software Review)Indexes = SCI-EXPANDED Timespan = All years
2. TS=((alpaca OR alpacas OR amphibian OR amphibians OR animal OR animals OR antelope OR armadillo OR armadillos OR avian OR baboon OR baboons OR beagle OR beagles OR bee OR bees OR bird OR birds OR bison OR bovine OR buffalo OR buffaloes OR buffalos OR “c elegans” OR “Caenorhabditis elegans” OR camel OR camels OR canine OR canines OR carp OR cats OR cattle OR chick OR chicken OR chickens OR chicks OR chimp OR chimpanze OR chimpanzees OR chimps OR cow OR cows OR “D melanogaster” OR “dairy calf” OR “dairy calves” OR deer OR dog OR dogs OR donkey OR donkeys OR drosophila OR “Drosophila melanogaster” OR duck OR duckling OR ducklings OR ducks OR equid OR equids OR equine OR equines OR feline OR felines OR ferret OR ferrets OR finch OR finches OR fish OR flatworm OR flatworms OR fox OR foxes OR frog OR frogs OR “fruit flies” OR “fruit fly” OR “G mellonella” OR “Galleria mellonella” OR geese OR gerbil OR gerbils OR goat OR goats OR goose OR gorilla OR gorillas OR hamster OR hamsters OR hare OR hares OR heifer OR heifers OR horse OR horses OR insect OR insects OR jellyfish OR kangaroo OR kangaroos OR kitten OR kittens OR lagomorph OR lagomorphs OR lamb OR lambs OR llama OR llamas OR macaque OR macaques OR macaw OR macaws OR marmoset OR marmosets OR mice OR minipig OR minipigs OR mink OR minks OR monkey OR monkeys OR mouse OR mule OR mules OR nematode OR nematodes OR octopus OR octopuses OR orangutan OR “orang-utan” OR orangutans OR “orang-utans” OR oxen OR parrot OR parrots OR pig OR pigeon OR pigeons OR piglet OR piglets OR pigs OR porcine OR primate OR primates OR quail OR rabbit OR rabbits OR rat OR rats OR reptile OR reptiles OR rodent OR rodents OR ruminant OR ruminants OR salmon OR sheep OR shrimp OR slug OR slugs OR swine OR tamarin OR tamarins OR toad OR toads OR trout OR urchin OR urchins OR vole OR voles OR waxworm OR waxworms OR worm OR worms OR xenopus OR “zebra fish” OR zebrafish) NOT (human OR humans or patient or patients))
3. 1 NOT 2
4. PMID=(0* or 1* or 2* or 3* or 4* or 5* or 6* or 7* or 8* or 9*)
5. 3 NOT 4
